# Immunotherapy augments the effect of 5-azacytidine on HPV16-associated tumours with different MHC class I-expression status

**DOI:** 10.1038/bjc.2011.428

**Published:** 2011-10-20

**Authors:** J Šímová, V Polláková, M Indrová, R Mikyšková, J Bieblová, I Štěpánek, J Bubeník, M Reiniš

**Affiliations:** 1 Department of Tumour Immunology, Institute of Molecular Genetics, v.v.i., Academy of Sciences of the Czech Republic, Videnska 1083, Prague 4 14220, Czech Republic

**Keywords:** 5-azacytidine, MHC class I downregulation, tumour chemoimmunotherapy, epigenetics, IL-12, CpG oligodeoxynucleotides

## Abstract

**Background:**

Epigenetic mechanisms have important roles in the tumour escape from immune responses, such as in MHC class I downregulation or altered expression of other components involved in antigen presentation. Chemotherapy with DNA methyltransferase inhibitors (DNMTi) can thus influence the tumour cell interactions with the immune system and their sensitivity to immunotherapy.

**Methods:**

We evaluated the therapeutic effects of the DNMTi 5-azacytidine (5AC) against experimental MHC class I-deficient and -positive tumours. The 5AC therapy was combined with immunotherapy, using a murine model for HPV16-associated tumours.

**Results:**

We have demonstrated 5AC additive effects against MHC class I-positive and -deficient tumours when combined with unmethylated CpG oligodeoxynucleotides or with IL-12-producing cellular vaccine. The efficacy of the combined chemoimmunotherapy against originally MHC class I-deficient tumours was partially dependent on the CD8^+^-mediated immune responses. Increased cell surface expression of MHC class I cell molecules, associated with upregulation of the antigen-presenting machinery-related genes, as well as of genes encoding selected components of the IFN*γ*-signalling pathway in tumours explanted from 5AC-treated animals, were observed.

**Conclusion:**

Our data suggest that chemotherapy of MHC class I-deficient tumours with 5AC combined with immunotherapy is an attractive setting in the treatment of MHC class I-deficient tumours.

Epigenetic modifications of the mammalian genome, including aberrant DNA methylation, represent tumourigenic events that are functionally equivalent to genetic changes (Jones and Baylin, 2007). Cellular pathways can be affected by ‘epigenetic’ agents, such as histone deacetylase and DNA methyltransferase inhibitors (DNMTi), which can reverse aberrant DNA methylation and/or histone acetylation in tumour cells. Their therapeutic benefit has been successfully tested in clinical trials and several compounds, including DNMTi 5-azacytidine (5AC) and 5-aza-2′-deoxycytidine (DAC), have been approved for clinical use (Mai and Altucci, 2009).

MHC class I downregulation on tumour cells in the course of their growth represents a frequent mechanism by which tumour cells can escape from the specific immune responses (reviewed by Garrido *et al*, 2010). Effective anti-tumour immunotherapy should thus be optimised to cope with MHC class I-deficient tumours (Bubenik, 2003; Reinis, 2010). Notably, procedures that lead to MHC class I upregulation on tumour cells have been examined to augment the efficacy of the immunotherapy. Various mechanisms, both reversible and irreversible, underlie the MHC class I downregulation. DNA methylation was found to be responsible for the MHC class I heavy chain gene inhibition (Nie *et al*, 2001; Serrano *et al*, 2001), while both the DNA methylation and histone acetylation changes were associated with inhibition of the antigen-presenting machinery (APM) gene expression (Seliger *et al*, 2006; Setiadi *et al*, 2007, 2008; Campoli and Ferrone, 2008; Khan *et al*, 2008; Manning *et al*, 2008; Adair and Hogan, 2009). Epigenetic agents can also induce (re)expression of a number of silenced genes encoding other immunoactive molecules, such as co-stimulatory molecules, adhesive ICAM-1 (CD54), NKG2D receptor and tumour-associated antigens (Tomasi *et al*, 2006; Fonsatti *et al*, 2007; Sers *et al*, 2009). The epigenetic agents can also sensitise tumour cells to apoptosis and facilitate their killing by cytotoxic T lymphocytes (Fulda and Debatin, 2006). Thus, the combination of chemotherapy with DNA methytransferase inhibitors and immunotherapy can be a promising therapeutic setting.

*In vivo* administration of epigenetic agents can influence tumour cell interactions with the immune system not only by affecting the tumour cells, but also by exerting their effects on immunocytes. The effects on immunocytes may be immunosuppressive. It has been shown that 5AC induced regulatory T cells by *FoxP3* expression activation (Lal *et al*, 2009). Negative effects of histone deacetylase inhibitors on dendritic cell maturation and antigen-presenting capacity have also been described (Nencioni *et al*, 2007, Kim and Lee, 2010). Moreover, histone deacetylase inhibitors decreased toll-like receptor-mediated activation of proinflammatory gene expression (Bode *et al*, 2007). The histone deacetylase inhibitors exert their anti-inflammatory effects by blocking the secretion of proinflammatory cytokines, such as TNF-*α*, IL-1*β*, IL-6 and IL-12 (Leoni *et al*, 2002). On the other hand, 5AC has been shown to induce differentiation of the myeloid-derived suppressor cells towards immunogenic antigen-presenting cells (Daurkin *et al*, 2010).

The aim of this study was to determine the *in vivo* effects of 5AC on expression of the MHC class I molecules and co-stimulatory molecules on tumour cells, using an animal model for MHC class I-deficient, HPV16-associated tumours (Bubenik, 2008). The question was how 5AC as the epigenetic agent can influence the anti-tumour immune responses *in vivo* and whether the treatment with epigenetic agents can be successfully combined with some immunotherapeutic protocols. We determined the additive/synergistic effects of 5AC with immunotherapy performed by the treatment with unmethylated CpG oligodeoxynucleotides (CpG ODN) or IL-12-producing cellular vaccines. Special attention was paid to the role of CTLs in 5AC-treated MHC class I-deficient tumour-bearing animals, as well as to the impacts of the 5AC treatment on the CpG ODN-induced activation of the immune system.

## Materials and methods

### Mice

C57BL/6 males, 2–4 months old, were obtained from AnLab Co., Prague, Czech Republic. The mice were housed in the animal facility of the Institute of Molecular Genetics AS CR. Experimental protocols were approved by the Institutional Animal Care Committee of the Institute of Molecular Genetics AS CR, Prague.

### Cell culture

MHC class I-positive cell line TC-1 was obtained by *in vitro* co-transfection of murine lung C57BL/6 cells with HPV16 *E6/E7* and activated human *Ha*-*ras* (G12V) oncogenes (Lin *et al*, 1996). TC-1/A9 (MHC class I-deficient) cell line (Smahel *et al*, 2003) was obtained from the TC-1 tumours developed in immunised mice. IL-12-gene-modified TC-1/IL-12 (231/clone 15) cells used for vaccination produced *in vitro* 40 ng IL-12/1 × 10^5^ cells ml^–1^ medium/48 h and were irradiated (150 Gy) before use (Indrova *et al*, 2006). All cell lines were cultured in RPMI 1640 medium supplemented with 10% fetal calf serum, L-glutamine and antibiotics.

### *In vivo* experiments

TC-1 or TC-1/A9 tumour cells were transplanted subcutaneously (s.c.) in a dose of 1 × 10^4^ into syngeneic mice. Mice were injected s.c. with 100 *μ*g of 5AC (Sigma, Saint Louis, MO, USA) in the vicinity of tumour transplantation on days 3, 7, 10, 14, 17, 21, 24 and 28. CpG ODN 1826 was injected s.c. on days 3 and 10. On day 4, mice were treated s.c. with 1 × 10^7^ 150 Gy irradiated TC-1/IL-12 cells. The mice (eight per group) were observed twice a week, and the number of mice with tumours, as well as the size of the tumours was recorded. All *in vivo* experiments were repeated at least twice with similar results. CpG ODN 1826 (5′-TCCATGACGTTCCTGACGTT-3′, phosphorothioate) (Gramzinski *et al*, 2001) was purchased from Genosys, Hradec Kralove, Czech Republic.

### *In vivo* depletion studies

*In vivo* depletion of NK1.1^+^, CD4^+^ and CD8^+^ cells was performed using monoclonal antibodies PK 136, GK 1.5 and 2.43, as described previously (Reinis *et al*, 2006). To deplete the effector cells, 0.1 mg of the antibody was i.p. injected into mice, during the first week, injections were given three times and in the following 2 weeks, mice received injections once a week. Depletion was verified by the staining of spleen cells with labelled antibodies and FACS analysis.

### Flow cytometry

Cell suspensions were prepared from tumours explanted from killed animals. Cells were further cultured for 7 days *in vitro* and subjected, together with original cell lines, to the FACS analysis as described previously (Mikyskova *et al*, 2005). In selected experiments, rIFN*γ* (50 U ml^–1^) was added into the culture medium 48 h before analysis. Cell surface MHC class I expression on tumour cells was determined using PE anti-H-2D^b^ (clone KH95) and PE anti-H-2K^b^ (AF6-88.5) antibodies. The following antibodies were used: PE anti-CD80 (16-10A1), PE anti-CD86 (B7-2) (GL1), PE anti-CD54 (ICAM-1) (3E2), PE anti-B7-H1 (CD274) (MIH5) and FITC anti-MHC class II I-A^b^ molecules (AF6-120.1). All cells were initially pre-incubated with anti-CD16/CD32 to determinate non-specific binding. Flow cytometry was performed using an LSR II flow cytometer (BD Biosciences, San Jose, CA, USA), 10 000 cells were counted. All antibodies used, including the relevant isotypic control, were obtained from BD Pharmingen (San Diego, CA, USA). For the phenotypic analysis of different populations of spleen cells, mice were killed (15 days after the TC-1/A9 transplantation and treatment with 5AC and CpG ODN 1826) and the suspension of spleen cells was prepared. After lysis of the erythrocytes with Tris–NH_4_Cl buffer, the expression of selected markers on spleen cells was analysed by flow cytometry. The following labelled antibodies were used: APC anti-CD45 (LCA, LY5), APC anti-CD11c (Integrin alpha_x_ chain) (HL3), APC anti-Gr-1 Ly-6G and Ly-6C (Rb6-8C5), FITC anti-CD11b (M1/70), FITC anti-CD4 (L3T4) GK1.5, PE anti-CD25 (IL-2-Receptor-Chain p55) (PC61), FITC anti-CD69 (H1.2F3), PE anti-NK1.1 (NKR-P1B and NKR-P1C) (PK136), FITC anti-CD19 (1D3) and PE anti-F4/80 (CIA3-1). As isotype controls, FITC-, APC- and PE-labelled antibodies of irrelevant specificity were utilised. All antibodies but anti-F4/80 (Biolegend, San Diego, CA, USA) were purchased from BD Pharmingen. For the tetramer assay, 100 000 spleen cells were counted. Cells were stained with PE tetramers containing mouse E7 (49–57) CTL epitope (Sanquin, Amsterdam, The Netherlands), followed by staining APC with anti-CD3e (145-2C11) and FITC anti-CD8a (53–6.7). In all experiments, samples from at least three mice per group were analysed.

### Real-time quantitative RT–PCR

Total RNA was extracted with High Pure RNA isolation kit (Roche, Basel, Switzerland). The amount of 1 *μ*g of RNA was reverse transcribed to cDNA using random hexamer primers from GeneAmp RNA PCR Core Kit (Applied Biosystems, Foster City, CA, USA) in a 20-*μ*l reaction volume at 42 °C for 30 min. Quantitation of PCR products was performed in 10 *μ*l of Lightcycler 480 SYBR Green I Master mix (Roche) using a real-time PCR lightcycler (Roche). DNA was denatured at 95 °C for 5 min; 45 cycles of denaturation at 95 °C for 25 s, annealing at 60 °C for 45 s, elongation at 72 °C for 1 min and incubation at 80 °C for 5 s. cDNAs were amplified with specific primers for *β*-actin, TAP-1, LMP-2, TAP-2, LMP-7, tapasin, IRF-1, IRF-8 and STAT-1. The list of the TAP-1/2, LMP-2/7 and reference genes and their primer sequences have been described elsewhere (Manning *et al*, 2008). The remaining PCR primer sequences are as follows: tapasin, 5′-GCTATACTTCAAGGTGGATGACC (forward) and TGCAAGACAGAGCAGTTCTGGG (reverse); IRF-1, 5′-GCCCGGACACTTTCTCTGATG (forward) and AGACTGCTGCTGACGACACACG (reverse); IRF-8, 5′-CGGGG CTGATCTGGGAAAAT (forward) and CACAGCGTAACCTCGTCTTC (reverse); STAT-1, 5′-TCACAGTGGTTCGAGCTTCAG (forward) and GCAAACGAGACATCATAGGCA (reverse); HPV16E6, 5′-GCAAGCAACAGTTACTGCGA (forward) and GTTGTCTCTGGTTGCAAATC (reverse); HPV16E7, 5′-ATGCATGGAGATACACCTAC (forward) and CGCACACAATTCCTAGTG (reverse). Fold changes in transcript levels were calculated using *C*_T_ values standardised to *β*-actin, used as the endogenous reference gene control. All samples were run in biological triplicates.

### Bisulphite modification and methylation-specific PCR

Treatment of DNA from TC-1/A9 cells with sodium bisulphite and methylation-specific PCR (MSP) analysis of the TAP-2, TAP-1 and LMP-7 promoter regions were performed by Bisulphite Epitect kit (Qiagen, Hilden, Germany) according to the manufacturer's protocol. In order to identify CpG islands within the promoter region of the antigen-processing genes, MSP analysis, performed with primers designed with the program METHPRIMER, was described elsewhere (Manning *et al*, 2008).

### Proliferation and ELISPOT assays

To determine the portion of the IFN*γ*-secreting spleen cells, an ELISPOT kit for detection of murine IFN*γ* (BD Pharmingen) was used. Spleen cells were cultured for 48 h and then placed to the wells of ELISPOT plates (concentration 5 × 10^5^ cells per well) for 24 h. The plates were then processed according to the manufacturer's instructions. Coloured spots were counted with CTL Analyser LLC (CTL, Cleveland, OH, USA) and analysed using the ImmunoSpot Image Analyser software.

For proliferation assay, splenocytes were resuspended at the concentration of 10^7^ cells ml^–1^ in PBS supplemented with 5% FCS and labelled with 5,6-carboxy-fluorescein diacetate succinimidyl ester (CFSE; Sigma) by incubation for 5 min in 37 °C and 5% CO_2_ at the final concentration of 2.5 *μ*M. Labelling was quenched with RPMI 1640 supplemented with 10% FCS and the cells were washed twice before culturing in flat-bottom 24-well plates (1.5 × 10^6^ ml^–1^). After CFSE staining, splenocytes were cultured alone or in the presence of immobilised anti-CD3 antibody (145-2C11; 1 *μ*l ml^–1^) and anti-CD28 (37.51; 1 *μ*l ml^–1^). For FACS analysis, anti-CD3e (145-2C11), CD4 (GK1.5), CD8a (53–6.7) antibodies were used to determine proliferation of CD3, CD8 and CD4-positive cells. Flow cytometry was performed using an LSR II flow cytometer (BD Biosciences), 10 000 cells were counted. All antibodies used, including the relevant isotope control, were obtained from BD Pharmingen.

### Statistical analysis

For statistical analyses of differences between the growth curves of tumours, the analysis of variance (Newman–Keuls and Tukey–Kramer tests) from NCSS, Number Cruncher Statistical System (Kaysville, UT, USA), statistical package was used. For statistical analysis of qPCR and ELISPOT assays, the Student's *t*-test was used. Differences between experimental and control samples with *P*<0.05 were considered to be statistically significant.

## Results

### Anti-tumour effects of 5AC on TC-1 and TC-1/A9 tumours are augmented by immunotherapy

**Figure 1:**
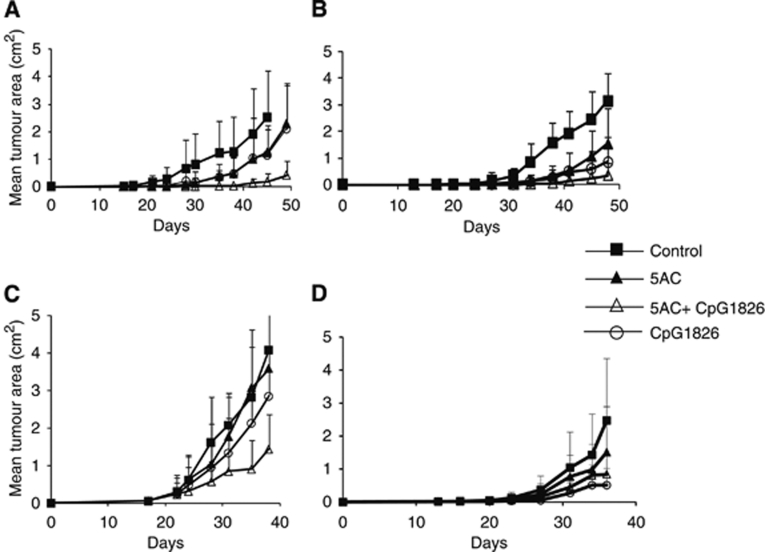
Tumour inhibitory effects of combined 5AC and CpG ODN 1826. Chemoimmunotherapy of the TC-1/A9 and TC-1 tumours. TC-1/A9 (**A**) and TC-1 (**B**) tumour cells were transplanted on day 0. In experimental groups, 5AC was repeatedly administered on days 3, 7, 10, 14, 17, 21, 24 and 28. CpG ODN 1826 was administered on days 3 and 10. Significant inhibition (*P*<0.05 determined by Newman–Keuls and Tukey–Kramer tests, eight mice per group were used for the experiments) was observed in all treated mice, as compared with the untreated controls. Combined therapy was significantly more effective as compared with monotherapies only. (**C**) CpG ODN and 5AC treatment started when the TC-1/A9 tumours became palpable with ∼1 mm in diameter. Significant inhibition was observed only after the combined therapy. (**D**) Treatment of TC-1 palpable tumours. The level of tumour growth inhibition by monotherapies was not different from the effects of the combined therapy.

**Figure 2:**
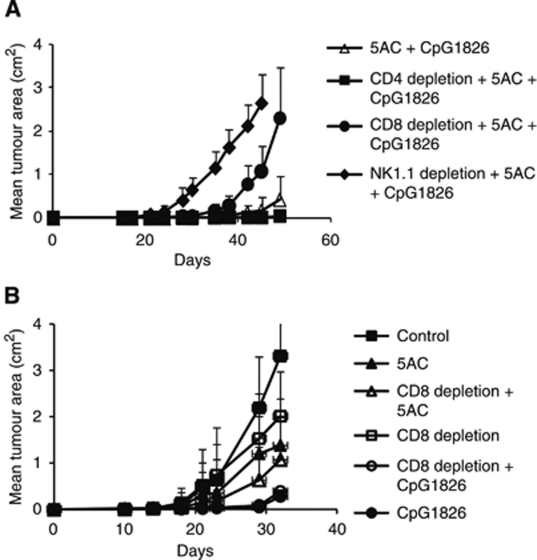
The impact of NK1.1^+^, CD4^+^ and CD8^+^ *in vivo* depletion on the 5AC and CpG ODN 1826 therapeutic effects against the TC-1/A9 tumours. (**A**) The efficacy of the combined 5AC and CpG ODN treatment of the TC-1/A9 tumours was significantly abrogated in mice with depleted CD8^+^ and NK1.1^+^ but not the CD4^+^ cells. (**B**) Tumour growth in mice subjected to the CpG ODN or 5AC monotherapies was not significantly changed upon CD8^+^ cell depletion, as compared with the treated-only controls. In all experiments, error bars show standard deviations. All experiments were repeated twice with similar results.

**Figure 3:**
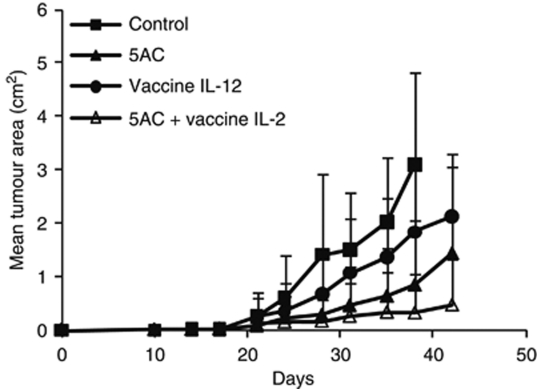
Tumour inhibitory effects of combined 5AC and TC-1/IL-12-producing cellular vaccine therapy of the TC-1/A9 tumours. TC-1/A9 (cells were transplanted on day 0). In experimental groups, 5AC was repeatedly administered on days 3, 7, 10, 14, 17, 21, 24 and 28; IL-12-producing cells were transplanted on day 4. Significant inhibition (*P*<0.05 determined by Newman–Keuls and Tukey–Kramer tests) was observed in all treated mice, as compared with the untreated control. The combined therapy was significantly more effective as compared with the 5AC treatment only.

The therapeutic effect of DNMTi 5AC against both TC-1/A9 and TC-1 tumours was demonstrated. Mice were transplanted with TC-1/A9 or TC-1 tumour cells and repeatedly treated with the DNA methytransferase inhibitor 5AC, administered intratumourally or into the vicinity of the site of tumour cell injection when the tumours were not yet palpable. As expected, significant inhibition of the tumour growth was observed in mice bearing both TC-1 and TC-1/A9 tumours (Figure 1). Further, the CpG ODN 1826 treatment significantly augmented the 5AC therapeutic effect. The effects of intratumoural administration of 5AC and CpG ODN 1826 and their combinations on the growth of palpable TC-1/A9 tumours were also investigated. In this setting, only the combination but not the 5AC or CpG ODN monotherapies significantly inhibited the tumour growth (Figure 1C). On the other hand, additive or synergistic effects were not observed in the therapy of the TC-1 tumours in which CpG ODN monotherapy significantly inhibited the tumour growth (Figure 1D). Since the MHC class I upregulation was observed on cells of growing TC-1/A9 tumours from 5AC-treated animals, we raised the question whether the role of the MHC class I-restricted mechanisms was increased in the immune response leading to the inhibition of the tumour growth. The role of CD4^+^, CD8^+^ and NK cells was assessed in the *in vivo* depletion experiments (Figure 2). Although the NK1.1^+^ cells remained critical for the tumour growth inhibition, the depletion of CD8^+^ cells also resulted in acceleration of the tumour growth in 5AC- and CpG ODN-treated animals, while the growth of the TC-1/A9 tumours in CpG ODN 1826 only-treated animals was not affected by the CD8^+^ cell depletion. These results indicate that 5AC therapy increased tumour cells’ sensitivity to CTL-mediated cytotoxicity. Further, the efficacy of the 5AC monotherapy, which led to MHC class I upregulation on tumour cells, was not dependent on the CD8^+^ cell population. This result suggests that besides the 5AC treatment, which increased the MHC class I expression on tumour cells, induction of immune response by immunotherapy was also crucial for the development or efficiency of the CD8^+^-mediated immunity. In the next series of *in vivo* experiments, the efficacy of combined therapy of MHC class I-deficient TC-1/A9 tumours with IL-12-producing vaccine (irradiated TC-1/IL-12 cell line) and 5AC was tested. As for the CpG ODN treatment, the combined therapy (i.e., treatments with 5AC on days 3, 7, 10, 14, 17, 21, 24, 28 and with TC-1/IL-12 on day 4) resulted in significantly more efficient inhibition of the tumour growth, as compared with monotherapy (Figure 3).

### *In vivo* and *in vitro* cell surface molecule modulation on tumour cells after the 5AC treatment

**Figure 4:**
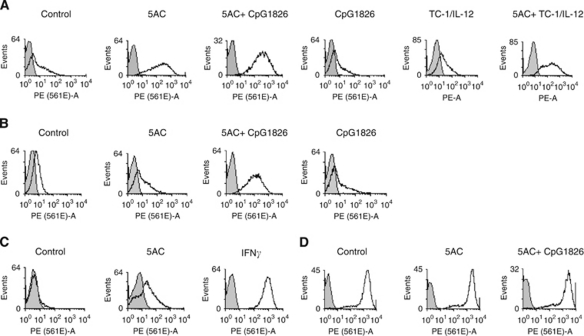
MHC class I expression on TC-1/A9 tumour cells. MHC class I expression (determined by using a mix of anti-H-2D^b^ and anti-H-2K^b^) was determined by the FACS analysis of tumour cells explanted from experimental and control animals, which underwent therapy either immediately after tumour cell transplantation (**A**) or when tumours became palpable (**B**). Control data of the TC-1/A9 cultured cells and treated *in vitro* with either 5AC or IFN*γ* (**C**) and of the explanted tumour cells from control and treated animals subsequently treated *in vitro* with IFN*γ* (**D**) are also shown. Representative results are presented; the statistical analysis of at least triplicate analyses is shown in Table 1.

**Figure 5:**
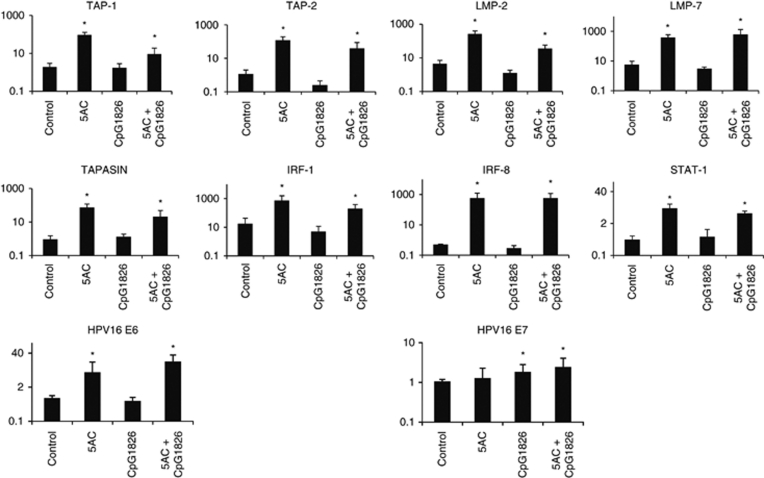
Upregulation of APM genes, IFN*γ* pathway components and E6/E7 oncogenes in TC-1/A9 tumours explanted from the 5AC- and/or CpG ODN-treated animals. Expression levels of selected APM genes, as well as *IRF-1*, *IRF-8* and *STAT-1* in TC-1/A9 tumour cells explanted from experimental and control animals. ^*^Denotes significant changes (*P*<0.05 determined in Student's *t*-test) as compared with the values from untreated animals. Biological triplicates were used for the analysis. In all experiments, error bars show standard deviations. Relative expression numbers represent the percentage of the *β*-actin expression levels.

**Figure 6:**
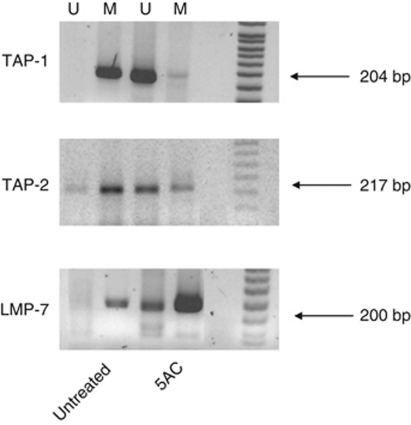
Methylation status of the APM gene regulatory sequences in the TC-1/A9 tumour cells from 5AC-treated and -untreated animals DNA from the TC-1/A9 explanted tumour cells from control 5AC-treated animals was bisulphite treated and subjected to the MSP analysis. The methylation status of the *TAP-1*, *TAP-2* and *LMP-7* promoter sequences was analysed. Bands in the lanes designated U represent the PCR products amplified from unmethylated DNA, bands from the M lanes represent the PCR products from methylated DNA.

We have assessed the level of the MHC class I and selected immunoactive molecules expression on the TC-1/A9 tumour cells excised from the tumour-bearing animals and cultured *ex vivo* and compared these levels with the expression level on the cells treated *in vitro* with the epigenetic agents or IFN*γ* (Figure 4 and Table 1). Indeed, the MHC class I expression on tumour cells from 5AC-treated animals was upregulated as compared with the tumour cells from untreated animals. Explanted tumour cells remained fully sensitive to the IFN*γ* treatment. We have also investigated the expression of other selected co-stimulatory molecules from the B7 family, as well as of CD54 (ICAM-1). The cell surface expression of CD80 was decreased in the tumour cells from the 5AC-treated animals, as compared with those from the untreated controls, while the B7-H1 molecules were moderately upregulated (a significant change was observed only in the CpG ODN/5AC-treated group). No significant changes were observed in CD54 expression (Table 1). Tumour cells remained CD86-, MHC class II- and B7-H2-negative after all treatments (data not shown). The results indicate that cell treatment with 5AC *in vivo* and *in vitro* results in a similar pattern of the monitored cell surface molecules. However, the MHC class I expression from explanted tumours was higher than that could be achieved upon the treatments of the tumour cell lines *in vitro*. As expected, the MHC class I upregulation induced by 5AC was associated with increased expression of APM genes (Figure 5). DNMTi effects on the APM and co-stimulatory/inhibitory gene expression *in vivo* in some tumour cells resemble the impacts of IFN*γ* on the expression of these genes. We have hypothesised that DNMTi can also act through the activation of the IFN*γ*-signalling pathway components and we therefore focused on the expression levels of the selected genes from this pathway, namely interferon responsible factors 1 (*IRF-1*) and 8 (*IRF-8*) and *STAT-1* in tumour cells. Indeed, the expression levels of these genes were increased in the cells from the 5AC-treated animals, as compared with the samples from the untreated animals or from mice after immunotherapy only. Increased sensitivity of tumour cells to the specific lysis could also be attributed to increased expression of tumour rejection antigens. Our analysis demonstrates the increased expression of both HPV16 E6 and E7 oncogenes. It is noteworthy that the TC-1 cell line has been engineered by transfection of plasmids carrying both these oncogenes. Therefore, analysis of their expression regulation is not relevant for understanding of the biology of cervical carcinoma cells. We have previously shown that upregulation of the APM gene expression upon *in vitro* treatment of the TC-1/A9 cells with the epigenetic agents is associated with DNA demethylation of the corresponding regulatory gene sequences (Manning *et al*, 2008). Here, we document a similar association after 5AC administration *in vivo* by the MSP analysis of the regulatory sequences of the selected APM genes (Figure 6). Explanted tumour cell retained their sensitivity to the IFN*γ* treatment. The expression of monitored genes was further increased upon 48 h *in vitro* treatment with 50 U ml^–1^ IFN*γ* (Supplementary Figure S1).

### Analysis of immunocyte populations and immune responses in 5AC-treated animals

**Figure 7:**
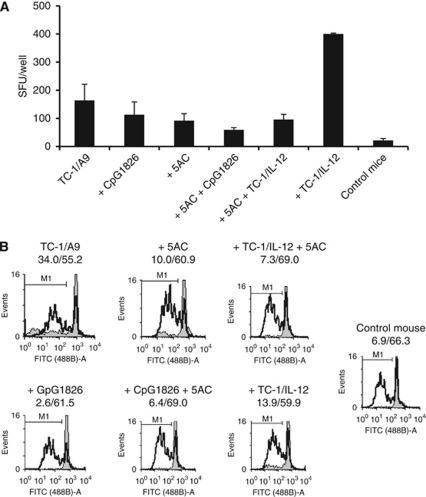
Analysis of spleen cell activation and immune responses in treated animals. Spleen cells were isolated from experimental and control groups 15 days after TC-1/A9 tumour cell transplantation and treatment commencement. (**A**) Spleen cells were subjected to the analysis of IFN*γ* production by ELISPOT assay. Biological triplicates were used for the analysis. In all experiments, error bars show standard deviations. In all experimental groups, the percentage of IFN*γ*-producing cells were significantly higher, as compared with healthy controls and lower, as compared with untreated tumour-bearing mice; 5AC treatment significantly decreased the percentage of IFN*γ*-producing cells from CpG- and TC-1/IL-12- treated, as well as untreated mice, respectively (*P*<0.05 determined in Students' *t*-test). (**B**) CD8^+^ spleen cell proliferation was determined by CFSE analysis. Representative data show CD8^+^ proliferation in unstimulated samples (grey histograms) and after CD3/CD28 mAb stimulation (white histograms). Numbers indicate the percentage of proliferating cells from unstimulated/stimulated samples.

We have monitored the immune responses after the treatment with 5AC combined with CpG ODN therapy or cell therapy with the IL-12-producing cells and, importantly, the impact of the 5AC administration on immune cells. The results are summarised in Figure 7 and Table 2. The percentage of IFN*γ*-producing spleen cells, as determined by ELISPOT assay, was significantly higher upon the 5AC and/or CpG ODN treatments, as compared with the healthy controls (Figure 7A). However, these levels were lower, as compared with the untreated tumour-bearing mice. Administration of 5AC also inhibited the activation effect of the IL-12-producing cellular vaccine.

The total numbers of the spleen cells were significantly lower in the 5AC-only treated animals (54 × 10^6^±14 × 10^6^), as compared with the tumour-bearing or healthy mouse controls (106 × 10^6^±34 × 10^6^ and 92 × 10^6^±19 × 10^6^, respectively). The total numbers of the spleen cells in all other experimental groups were not significantly different from the control groups. Although the percentage of proliferating cells in spleens were lower in all treated mice, as compared with tumour/bearing untreated mice, the capacity of the CD8^+^ spleen cells to proliferate upon CD3/CD28 activation was not significantly impaired (Figure 7B). Similar results as for the CD8^+^ cells were obtained for the CD4^+^ spleen cells (data not shown).

Detailed analysis of the spleen cell populations in 5AC- and/or CpG ODN-treated and untreated tumour-bearing and control mice is presented in Table 2. This analysis documents that 5AC treatment did not influence the increased proportion of activated (CD69^+^) T and B lymphocytes, as well as of NK cells induced by the CpG ODN treatment. As expected, CpG ODN increased the numbers of matured dendritic cells (CD11c^+^/CD86^+^/MHC class II^+^) and also the expression of B7-H1. 5AC had no effect on these numbers. In agreement with the *in vivo* therapeutic data showing that the CD8^+^ population had a role in the inhibition of the tumour growth only when 5AC and CpG were used in combination, the increased specific CD8^+^ spleen cell population recognising E7 antigen was documented by the tetrameric assay only in the spleens from 5AC and CpG ODN-treated animals. Collectively, this analysis reveals that the 5AC treatment does not dramatically influence the proportion of particular cell populations or their activation status, as well as the changes induced by CpG ODN.

## Discussion

DNMTi, such as 5AC, display a strong potential to be used as anti-tumour chemoterapeutics. Since they have been described to increase immunogenicity of tumour cells, as well as their sensitivity to the cytotoxic cells, they are attractive candidates for combination chemoimmunotherapy. Two studies, including ours (Manning *et al*, 2008; Setiadi *et al*, 2008), have recently documented MHC class I molecule upregulation on MHC class I-deficient TC-1/A9 tumour cells after DNMTi or HDACi treatments *in vitro* and, subsequently, after the treatment, these cell became sensitive for specific lysis by CTLs. Our aim in this study was to optimise the therapeutic protocols based on immunotherapy combined with DNMTi treatment, using the same model for MHC class I-deficient tumours. Previously, we have demonstrated that CpG ODN can inhibit the tumour growth of tumours with a different MHC class I cell surface expression status (Reinis *et al*, 2006). Similarly, the therapeutic effect of the IL-12-producing cellular vaccine was demonstrated in the treatment of the minimal residual tumour disease after chemotherapy (Indrova *et al*, 2003, 2008; Bubenik 2008).

In this study, we have shown the synergistic/additive effects of DNMTi treatment with non-specific immunotherapy using CpG ODN or cellular vaccine producing IL-12. Our data indicate that the *in vivo* treatment modulates immunogenicity of the TC-1/A9 tumour cells, since the *in vivo* cell depletion study revealed induction of CD8^+^ cell-dependent mechanisms in protective immune responses against these tumours. It is noteworthy that the CD8^+^ cell dependence of the therapeutic effect was not observed after 5AC monotherapy but only after combined treatment with CpG ODN. This result suggests that for maximal therapeutic effects, tumour cell sensitisation to immune responses by convenient chemotherapy with epigenetic agents should be combined with activation of the immune responses by immunotherapy. The *in vivo* depletion experiments revealed that the tumour growth in both 5AC-treated and untreated animals was strongly controlled by the NK1.1^+^ cells. This result documents the role of innate immunity against tumours regardless of their MHC class I expression status. Indeed, we have previously shown, using the TC-1 and TC-1/A9 models, that NK1.1^+^ cell population is an important player controlling the early phases of the parental, MHC class I-positive, TC-1 tumour growth (Simova *et al*, 2004; Reinis *et al*, 2006).

The additive effect of 5AC and CpG ODN administration was surprisingly not observed on palpable TC-1 tumours. The possible explanation might be that CpG ODN monotherapy was more effective against more immunogenic TC-1 tumours than against TC-1/A9 tumours so that it was difficult to see the increased efficacy of combined therapy in our experimental setting.

The phenotypic analyses showed significant MHC class I upregulation on the explanted TC-1/A9 tumour cells upon *in vivo* 5AC treatment. 5AC administration increased the expression of a number of APM genes (*TAP-1*, *TAP-2*, *LMP-2*, *LMP-7*, *Tapasin*). Interestingly, the MHC class I cell surface expression levels after *in vivo* administration of 5AC were higher, as compared with the expression levels achieved upon the *in vitro* treatment of the TC-1/A9 cells. This could be attributed to repeated treatments with 5AC or to the additive effects of endogenous cytokines in the tumour microenvironment, as the MHC class I expression tends to increase even in the tumours from mice that were not subjected to any therapy (Mikyskova *et al*, 2005).

Both *in vitro* and *in vivo* treatment with 5AC induced the expression of the APM and other genes, which are inducible by IFN*γ*. 5AC treatment combined with CpG ODN (in 5AC only-treated mice, the upregulation was not significant) moderately (much less than can be seen upon *in vitro* IFN*γ* treatment) increased the expression of the B7-H1-negative regulator on tumour cells, which is known to be regulated through the IFN*γ*-inducible IRF-1 factor (Lee *et al*, 2006). Further, it is also known that the *IRF-8* gene is frequently epigenetically silenced in a number of tumours and that DNMTi can increase tumour cell sensitivity to apoptosis through upregulation of IRF-8 (Fulda and Debatin, 2006). Therefore, we have decided to select for monitoring, besides *STAT-1*, the *IRF-1* and *IRF-8* gene expression upon the treatment with 5AC. Our data indicate that expression of these crucial players in the IFN*γ*-signalling pathway is increased in tumour cells from the 5AC-treated animals. Although more studies have to be done, this observation suggests that both, direct demethylation of the corresponding regulatory sequences of the upregulated genes, as well as upregulation of the critical components of the IFN*γ*-signalling pathway can take place in the modulation of the MHC class I and, or co-stimulatory or regulatory molecules.

Immunomodulatory effects of the hypomethylating agents can also be mediated by their effects on immune cells. Thus, it was important to assess how the *in vivo* administration of these agents influenced subsequent immunotherapy and anti-tumour immune responses upon non-specific immunotherapy. The results demonstrate that the 5AC treatment in our experimental settings can display adverse effects on the immune system, since the number of spleen cells was lower as compared with the 5AC-untreated controls. The 5AC treatment also decreased the percentage of the IFN*γ*-producing spleen cells in the tumour-bearing animals. However, the proliferative capacity and the proportion of particular spleen cell populations of the spleen cells remained unaffected. Also, 5AC also did not alter the numbers of activated T, B and NK cells induced by CpG ODN. Importantly, the synergistic effect of combined immunochemotherapy was observed on the induction of specific anti-E7 immunity. We have concluded that the potential 5AC adverse effects on the immune system were not an obstacle for an effective combination treatment with immunotherapy. Since chemo- or immunotherapy can also induce negative regulators of the immune responses, the therapeutic efficacy could be increased by combining the treatment with anti-immunosuppressive treatments.

Taken together, our data document that chemotherapy of MHC class I-deficient tumours with DNMTi combined with non-specific immunotherapy is a promising therapeutic setting against MHC class I-deficient tumours, although both positive and detrimental effects of DNMTis have to be considered and the immunotherapeutic settings have to be optimised.

## Figures and Tables

**Table 1:** Expression of MHC class I and co-stimulatory molecules on explanted TC-1/A9 tumour cells

	**Fluorescence intensity (Gmean)**			
**Group of mice**	**MHC class I**	**CD80**	**B7-H1**	**CD54**
Control	14.1±6.9	619.2±76.4	5.6±1.1	7.3±2.4
5AC	189.9±71.1^*^	413.2±244.4	12.0±7.7	8.6±6.0
5AC + CpG1826	196.0±50.0^*^	279.0±151.1^*^	10.9±3.4^*^	6.5±3.1
CpG1826	10.9±6.2	739.3±163.3	6.1±1.0	5.9±0.5

Abbreviations: MHC=major histocompatibility complex; 5AC=5-azacytidine.

^*^P<0.05 as compared with control group.

**Table 2:** Phenotypic characterisation of spleen cells from mice 15 days after TC-1/A9 transplantation and treatment with 5AC and/or CpG ODN 1826

	**% Positive CD45^+^ spleen cells from mice treated with**				
**Marker expression**	**Control TC-1/A9**	**5AC**	**5AC + CpG 1826**	**CpG 1826**	**Control mice**
CD8	13.3±2.4	15.7±0.5	13.1±2.4	11.8±0.6	14.8±1.4
CD8/CD69	0.9±0.2	0.9±0.0	2.5±0.1^*^	2.5±0.5^*^	0.8±0.0
CD4	17.5±1.6	20.4±1.3	17.1±2.3	17.3±1.2	21.7±2.8
CD4/CD69	3.1±0.2	3.9±0.5	6.0±1.9^*^	5.6±0.5^*^	2.8±0.1
NK1.1	7.4±0.4	6.5±0.5	18.1±2.4^*^	13.7±1.5^*^	4.9±0.2
NK1.1/CD69	3.8±0.7	3.7±0.3	10.3±1.6^*^	7.2±0.3^*^	2.2±0.2
CD19	58.7±4.2^*^	69.0±2.4^†^	65.6±5.3^†^	72.1±3.8^†^	76.1±4.0^†^
CD19/CD69	3.3±0.6	4.8±0.2	8.0±0.8^*^	12.5±1.6^*^	4.4±0.6
Gr-1	7.9±1.9	5.6±0.6	12.5±0.3^*^	11.6±1.6^*^	9.3±0.3
Gr-1/CD11b	3.0±0.8	2.8±0.5	4.0±0.9	4.9±1.1^*^	3.0±0.3
F4/80	10.3±1.5^§^	10.3±0.8^§^	14.0±2.8^§^	10.3±1.8^§^	6.3±0.8
CD11c	11.2±0.2^**^	9.5±0.8^**^	6.5±0.5	6.7±1.1	7.0±0.8
CD45/B7-H1	3.2±0.6	4.8±0.2	8.0±0.8^*^	12.5±1.6^*^	4.4±0.6
CD86/MHC II	7.1±0.6	7.1±1.0	14.3±2.4^*^	16.1±1.8^*^	6.5±1.1
CD86/MHC II (in CD11c^+^ population)	50.6±4.7	53.1±1.8	76.4±4.7^*^	77.2±2.1^*^	51.6±5.8
Tetramer H2-D^b^/E7 (in CD8^+^ population)	0.17±0.03	0.2±0.02	0.36±0.06^***^	0.17±0.06	0.18±0.04

Abbreviations: MHC=major histocompatibility complex; ODN=oligodeoxynucleotides; 5AC=5-azacytidine.

Data from at least three mice were used for analysis.

^*^P<0.05 as compared with: control TC-1/A9, treated with 5AC, control mice.

^†^*P*<0.05 as compared with: control TC-1/A9.

^§^*P*<0.05 as compared with: control mice.

^**^P<0.05 as compared with: treated with 5AC+CpG 1826, CpG 1826, control mice.

^***^P<0.05 as compared with: 5AC, CpG1826, control TC-1/A9, control mice.
