# Supplementary figures and images for: Immunotherapy augments the effect of 5-azacytidine on HPV16-associated tumours with different MHC class I-expression status
Source: Br J Cancer. 2011 Oct 20;105(10):1533–41. doi: 10.1038/bjc.2011.428 (PMC3242529; doi:10.1038/bjc.2011.428)

## Slide 1
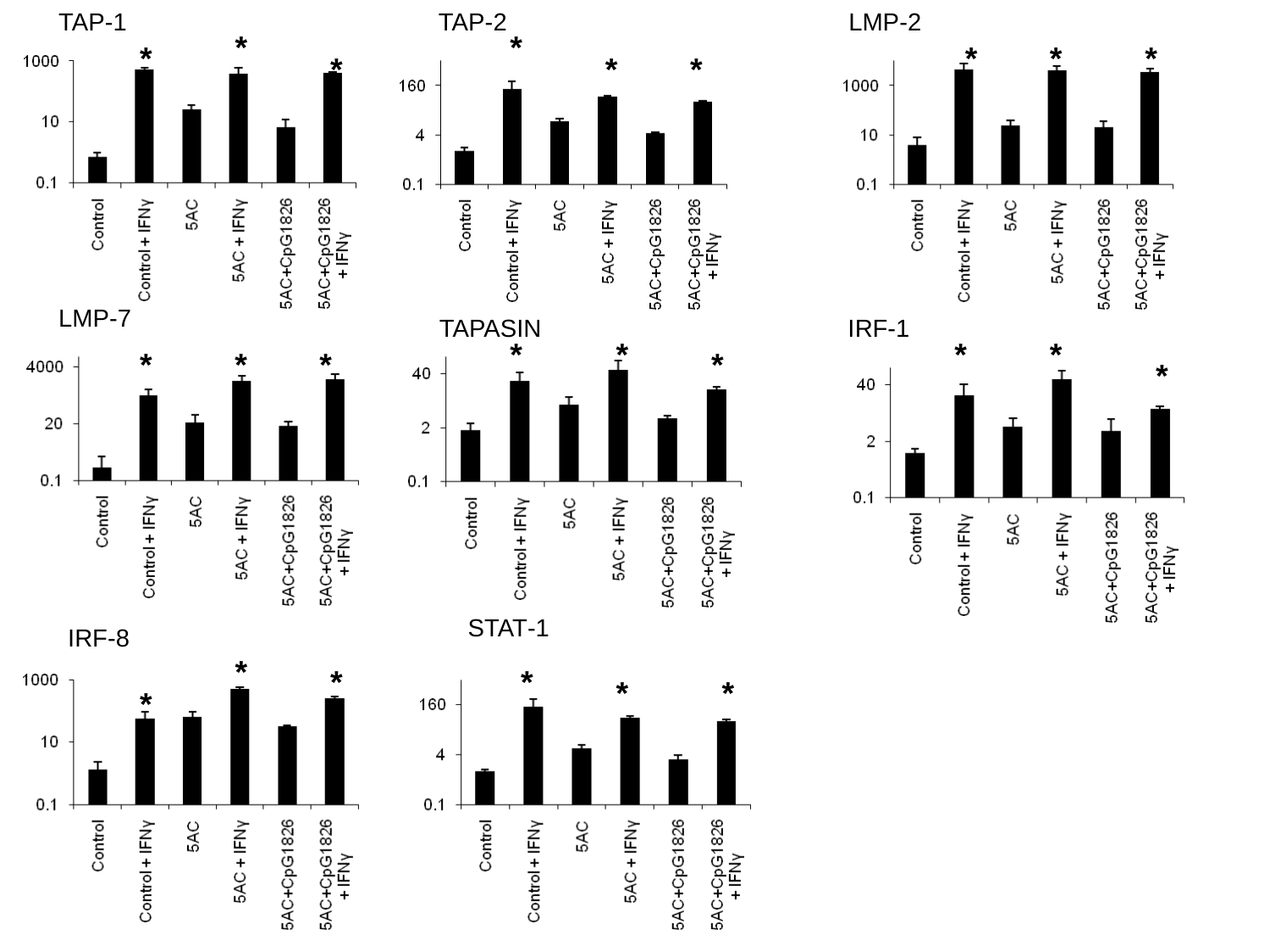

TAP-1
TAP-2
LMP-2
*
*
*
*
*
*
*
*
*
LMP-7
TAPASIN
IRF-1
*
*
*
*
*
*
*
*
*
STAT-1
IRF-8
*
*
*
*
*
*

Supplement: Supplementary Figure S1 [file bjc2011428x1.ppt]
