# Supplementary material for: Immunotherapy augments the effect of 5-azacytidine on HPV16-associated tumours with different MHC class I-expression status
Source: Br J Cancer. 2011 Oct 20;105(10):1533–41. doi: 10.1038/bjc.2011.428 (PMC3242529; doi:10.1038/bjc.2011.428)
Supplement: Supplementary Figure Legend [file bjc2011428x2.doc]

**Supplementary data**

**Figure S1**

Explanted tumour cell retain their sensitivity to IFNγ. Upregulation of APM genes and IFNγ pathway components in TC-1/A9 tumours explanted from the 5AC-treated (with or without CpG ODN 1826 addition) and control animals upon additional *in vitro* cultivation with IFNγ was assessed. *denote significant changes (P<0.05 determined in Student´s t test) as compared to the values from the corresponding IFNγ-untreated cells. Biological triplicates were used for the analysis. In all experiments, error bars show standard deviations. Relative expression numbers represent the percentage of the β-actin expression levels.
